# Compensation for the Decoherence Effect in Heterodyne Detection of Rough Targets and a Target Vibration Characteristic Measurement System

**DOI:** 10.1038/s41598-020-62966-0

**Published:** 2020-04-08

**Authors:** Changqing Cao, Xiyuan Su, Yutao Liu, Xiaodong Zeng, Zhejun Feng, Jingshi Shen, Ting Wang, Xu Yan

**Affiliations:** 1 School of Physics and Optoelectronic Engineering, Xidian University, 2 South Taibai Road, Xian, 710071 China; 2 Shandong Institute of Space Electronic Technology, Yantai, 264670 China

**Keywords:** Applied optics, Optoelectronic devices and components

## Abstract

In practical applications of signal detection, the roughness of a target surface significantly affects detection efficiency. In this paper, we propose a signal processing method that improves the sensitivity of a detection system by up to 100 times. In experiments, the target vibration measurement system successfully captured an automotive vibration power spectrum using the proposed signal processing method. This technology opens a new avenue for development in the field of rough surface target detection and recognition.

## Introduction

Heterodyne receivers offer superior sensitivity and selectivity because they use intermediate frequency (IF) amplifiers, which can easily obtain high gain. Heterodyne receivers have been widely used to detect weak signals in wireless communication systems, such as radio, TV, and radar receivers. In previous works, optical heterodyne technology has been applied to synthetic aperture lidar (SAL)^1,2^. Research on SAL has mainly focused on imaging technology^3,4^, phase compensation technology^5^, signal processing technology^6^, linear frequency hopping technology, and optical heterodyne detection^7–9^. In such applications, target information can be extracted directly from the IF signal (amplitude, frequency, or phase). In recent years, optical heterodyne technology has been developed into a key technology for target recognition, in an important technical breakthrough of combining rough target optical heterodyne detection with laser Doppler technology.

However, the sensitivity advantages of optical heterodyne receivers are not significant, with laser linewidth, atmospheric turbulence, and other aberrations affecting heterodyne detection performance^10,11^. It is known that optical heterodyne detection extracts information by matching a local oscillator (LO) light wave with a signal light wave. This LO light and signal light matching includes phase matching, amplitude matching, and polarisation matching. Wavefront matching between signal light and local light is very difficult, and significantly affects signal to noise ratios^12–16^.

Besides system parameters, the performance of a coherent detection system heavily depends on the characteristics of a target surface. In a coherent laser detection system, the return signal from a diffuse scattering surface are subject to the effects of phase fluctuation (the ‘decoherence effect’^17–19^), which can severely degrade heterodyne system performance^20–25^.

Many practical heterodyne detection systems use a single photodiode (PD). Because the wavelength of a laser is commonly on the order of micrometres, the surface of most practical target cannot be considered a perfect plane, which means that the target surface is rough. For a rough target (diffuse), the wavefront of the laser return is modulated randomly by the target surface. In other words, the wavefront fluctuation of the received wave will be affected by random variations the optical path length between the sensor and target surface scatterer. Such wavefront fluctuation can introduce two problems.

First, the random phase can mix with the signal phase, resulting in difficulty in extracting the element of the signal phase over the detector’s sensitive area. Owing to fluctuations in the laser echo wavefront, IF signals generated by different elements of the detector may cancel each other out, which can make IF signal measurement more difficult. Receiver system sensitivity degradation cannot be improved significantly by increasing IF amplifier gain based on the zero-mean value property of heterodyne signals.

Second, IF signal represents the integration of photocurrents^26,27^. Despite several studies on the decoherence effect, the compensation techniques that have been developed have made little progress toward its resolution in optically active heterodyne systems.

## Methods and experiments

In this paper, we introduce a novel orthogonal signal processing method to compensate for decreases in heterodyne detection sensitivity caused by the decoherence effect. Additionally, a novel vehicle recognition technology based on vibration signal characteristics is proposed and an automobile vibration measurement experiment is presented. In the proposed orthogonal signal processing method, we compensate for the decoherence effect in the electric domain. This method can be implemented easily using mature electronic techniques, which is a major advantage in terms of practicality.

**Figure 1:**
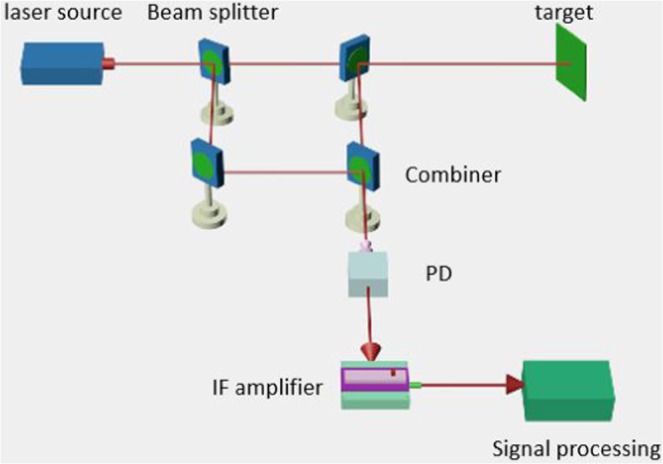
Schematic of a heterodyne detection system for rough target detection.

The principles of optical heterodyne detection based on laser returns from a target are illustrated schematically in Fig. 1.

**Figure 2:**
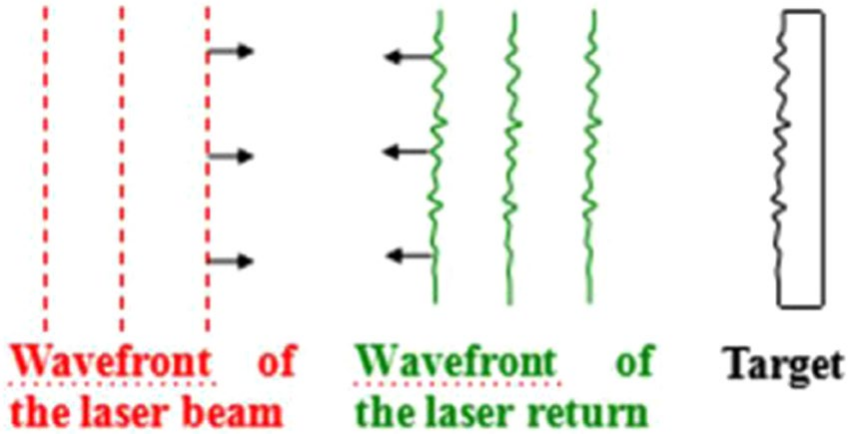
Modulation effect on the laser return wavefront by a rough target surface.

The laser beam illuminates the target and part of the light is scattered and returns to the receiver, where it mixes with the LO in the heterodyne receiver. Typically, the phase and amplitude of the laser beam are both modulated by the rough target surface. However, the influence of phase modulation on heterodyne performance is much greater than that of amplitude modulation. Thus, in this study, we only considered phase modulation by the target surface. If the surface of the target is rough (diffuse), then the wavefront of the laser returns will be modulated, as depicted in Fig. 2.

This spatial phase modulation can significantly decrease the sensitivity of the receiver. For convenience, we assume that the laser output and LO beams have linearly polarised plane wavefronts. Therefore, their electric fields near the sensitive area of the PD can be described as follows:

1 $${E}_{L}(r,t)={E}_{L}\,\cos [{\omega }_{L}t+{\varphi }_{L}],$$

2 $${E}_{S}(r,t)={E}_{S}\,\cos [{\omega }_{S}t+{\varphi }_{S}+{\varphi }_{S}(r)],$$

where *E*_*L*_*(r, t)* and *E*_*s*_*(r, t)* are the optical fields of the LO and signal, respectively, *φ*_*L*_ and *φ*_*s*_ are the constant phases, and *φ*_*s*_*(r)* denotes the modulation effect of the rough surface.

Generally, the height fluctuation of a rough surface is considered to follow a Gaussian distribution with a mean of zero. Therefore, the probability density function of *φ*_*s*_(*r*) can be described as shown in Eq. (3):

3 $$p(\phi )=\frac{1}{\sqrt{2\pi }\sigma }\exp \left[-\frac{{\varphi }^{2}}{2{\sigma }^{2}}\right].$$

As the wavelength of light waves is on the order of micrometres, fluctuations caused by rough surfaces are much greater than the wavelength, i.e. *σ*» 2π. Thus, *p*(*φ*) is approximately constant and *φ* follows a uniform distribution.

4 $$P(\varphi )=\{\begin{array}{c}\frac{1}{2\pi },\,-\pi  < \varphi  < \pi \\ 0,\,{\rm{otherwise}}\end{array}$$

The photocurrent generated by the PD can be written as:

5 $$d{i}_{IF}(t)=R{E}_{S}{E}_{L}\,\cos [\Omega t+{\varphi }_{S}-{\varphi }_{L}+{\varphi }_{S}(r)],$$

where Ω = *ω*_*s*_ *− ω*_*L*_ is the intermediate frequency (IF), and *R* is the responsivity of the photodetector. The total photocurrent generated by PD is obtained by integrating *di*_*IF*_ over the sensitive area of the PD, as in Eq. (6):

6 $${i}_{IF}(t)={\iint }_{A}d{i}_{IF}=C{\iint }_{A}\cos [\Omega t+{\varphi }_{S}(r)]ds,$$

where A is the area of the PD, *C* = RE_s_E_L_, and *φ*_*s*_ − *φ*_*L*_ is considered to be zero.

Equation (6) indicates that an IF signal can be considered as a superposition of several random components, implying that the IF signals generated by different elements of the detector can cancel each other out based on random phase fluctuations. IF signal should follow zero-mean Gaussian distribution according to the central limit theorem, assuming that the random fluctuations are independent^28^. Thus, we have

7 $$\langle {i}_{IF}(t)\rangle =0,$$

where 〈•〉 denotes an average over the ensemble of realisations.

This indicates that the amplitude of the useful signal, as measured according to the output of the heterodyne receiver, is equal to zero, which makes this signal difficult to detect. It is clear that increasing the gain of the IF amplifier cannot resolve this issue if 〈i_*IF*_(*t*)〉 = 0. To compensate for the degradation of the IF signal caused by the decoherence effect, the influence of *φ*_*s*_(*r*) on the IF signal must be eliminated.

Two methods have been developed to solve this problem^29^. In the first method, a single speckle grain is received by spatially filtering returning light. However, this can result in significant reductions in the magnitude of detected light, thereby reducing the detection sensitivity, making this method unsuitable for long-distance detection. In the second method, a photodetector array, such as a high-speed camera, is used to receive returning light. In this case, each pixel receives a single speckle grain, and the signals from each pixel are then superposed using an appropriate electric signal processing technique. This method does reduce the detected light level, but it is not a practical solution owing to the dual constraints of system cost and complexity.

Real detection targets such as building surfaces, different types of ground materials, and different types of coatings or material surfaces typically fit the two-dimensional random rough surface model with specific probability density distributions. Surface undulations in such targets represent rough targets for light waves. However, when using microwaves, such surfaces may appear to be smooth.

We used the Monte Carlo method for rough surface modelling in this study. Monte Carlo simulation^30–33^ is a statistical method that uses a power spectrum in the frequency domain as a filter, and then filters the results of inverse fast Fourier transforms, which measure wavelength as a unit. In our work, random rough surfaces were simulated by using the root mean square (*δ*) and correlation length (L) as main parameters.

The roughness of the root mean square is a target surface feature parameter that can be used to indicate target surface roughness, as shown in Eq. 8:

8 $$\delta =\sqrt{E[{h}^{2}(x)]-{\{E[h(x)]\}}^{2}}=\sqrt{{\int }_{-\infty }^{\infty }{h}^{2}p(h)dh-{[{\int }_{-\infty }^{\infty }hp(h)dh]}^{2}}$$

This parameter can be calculated numerically using specific calculation steps to select an appropriate interval. If the discrete interval is *x*, *N* is the number of sampling points. According to empirical laws, Δ*x* ≤ 0.1 *λ*, where *λ* is the incident wavelength; thus, the discrete value of *h*(x_i_) can be numerically calculated, as shown in (9):

9 $${\delta }^{2}=\frac{1}{N-1}[\mathop{\sum }\limits_{i=1}^{N}{({h}_{i})}^{2}-N{(\bar{h})}^{2}]$$

where

$$\bar{h}=\frac{1}{N}\mathop{\sum }\limits_{i=1}^{N}{h}_{i}.$$

To represent the roughness of a surface accurately, we must introduce a correlation function. The physical meaning of this parameter is the degree of association between two points on a rough surface. First, an autocorrelation function is defined, as in Eq. (10):

10 $$G(R)=E[h(x)h(x+R)],$$

where *G(0)* = *δ*^2^.

This can be normalised as shown in Eq. (11):

11 $$\rho (R)=\frac{E[h(x)h(x+R)]}{{\delta }^{2}},$$

where *R* represents the distance between two points on a rough surface, and *δ*^2^ represents the root mean square of the height of the surface.

For a better understanding of the decoherence effect, we used a Gaussian random rough surface model based on the Monte Carlo method as a target and simulated two-dimensional wavefront echoes from surfaces with different roughness values. We assumed that the two-dimensional random rough Gaussian lengths in the *X* and *Y* directions were *L*_*x*_ and *L*_*y*_, respectively, and that the interval sampling points were M and N, denoting the distance between two adjacent points, Δx and Δy, respectively. Thus, each point height on a rough surface can be expressed as shown in Eq. (12)^34,35^.

12 $$f({x}_{m},{y}_{m})=\frac{1}{{L}_{X}{L}_{Y}}\mathop{\sum }\limits_{{m}_{k}=-M/2+1}^{M/2}F({k}_{{m}_{k}},{k}_{{n}_{k}})\exp [i({k}_{{m}_{k}}{x}_{m}+{k}_{{n}_{k}}{y}_{n})],$$

where *x*_*m*_ = *n*Δx, *x*_*n*_ = *n*Δy, *m* = *−M/*2 + 1, …, *M*/2, *n* = −*N*/2, and

13 $$F({k}_{{m}_{k}},{k}_{{n}_{k}})=2\pi {[{L}_{x}{L}_{y}S({k}_{{m}_{k}},{k}_{{n}_{k}})]}^{1/2}\times \{\begin{array}{c}\frac{[N(0,1)+iN(0,1)]}{\sqrt{2}},{m}_{k}\ne 0,M/2;{n}_{k}\ne 0,N/2\\ N(0,1),{m}_{k}\ne 0,M/2;{n}_{k}\ne 0,N/2\end{array}$$

The Gaussian power spectral density for a two-dimensional, random rough Gaussian surface can then be written as shown in Eq. (14):

14 $$S({k}_{x},{k}_{y})={\delta }^{2}\frac{{l}_{x}{l}_{y}}{4\pi }\exp (-\frac{{k}_{x}^{2}{l}_{x}^{2}+{k}_{y}^{2}{k}_{y}^{2}}{4}),$$

where $${k}_{mk}=2\pi {m}_{k}/{L}_{x},$$ and $${k}_{nk}=2\pi {n}_{k}/{L}_{y}$$.

**Figure 3:**
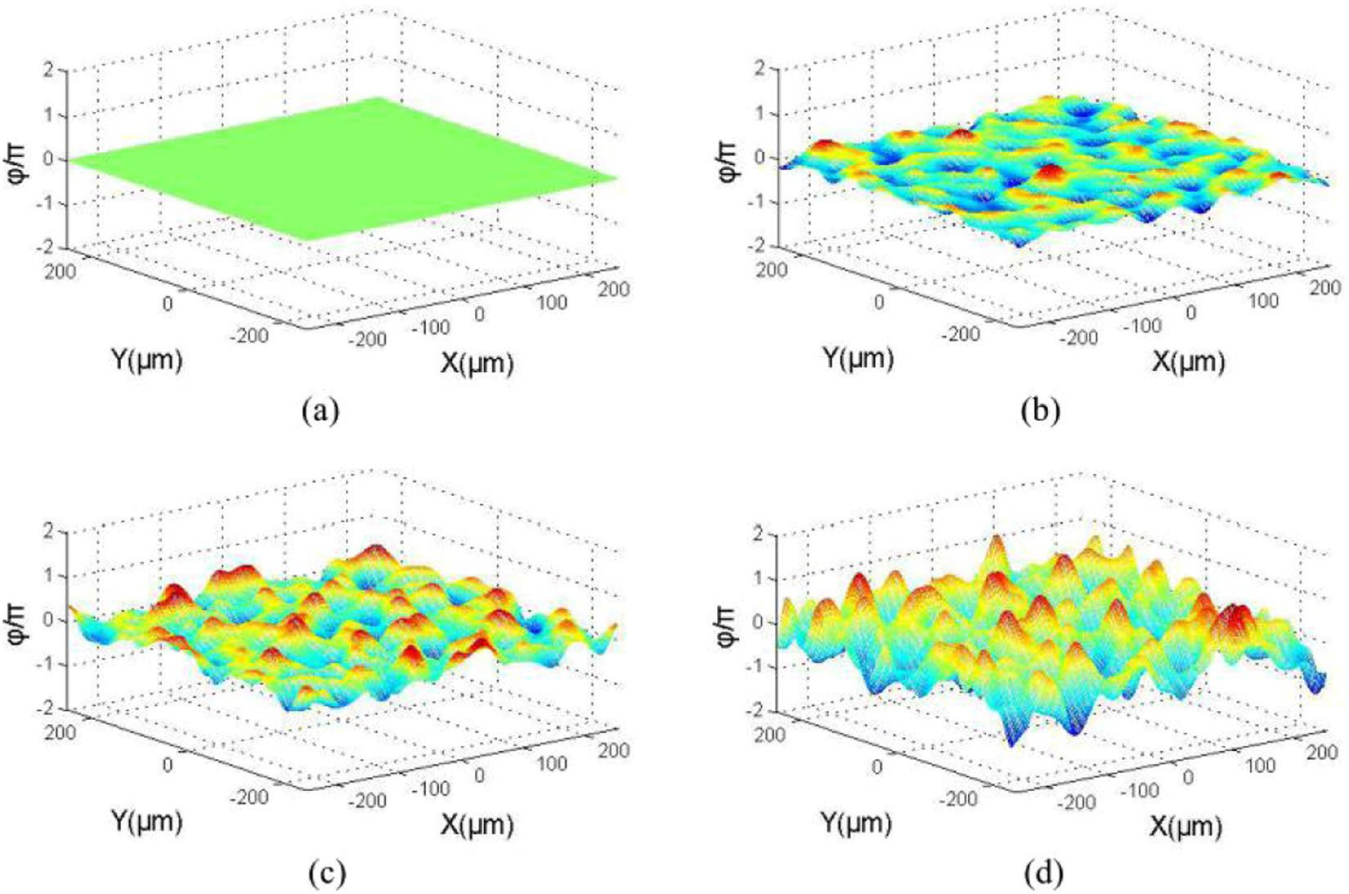
Four two-dimensional wavefronts of the echo, based on simulation, with the same coherence length and different degrees of roughness. In (**a**) *δ* = 0 *λ*, (**b**) *δ* = 0.1 *λ*, (**c**) *δ* = 0.2 *λ*, and (**d**) *δ* = 0.4 *λ*.

By using Eqs. (12), (13), and (14), a numerical simulation result for a two-dimensional, random rough Gaussian surface can be obtained. There are four, two-dimensional wavefronts of echoes with the same coherence length and different roughness values in anthe optical heterodyne detection system, as illustrated in Fig. 3.

Because the wavefronts have the same coherence length, a large value of root mean squared height indicates a greater degree of fluctuation in a rough surface.

**Figure 4:**
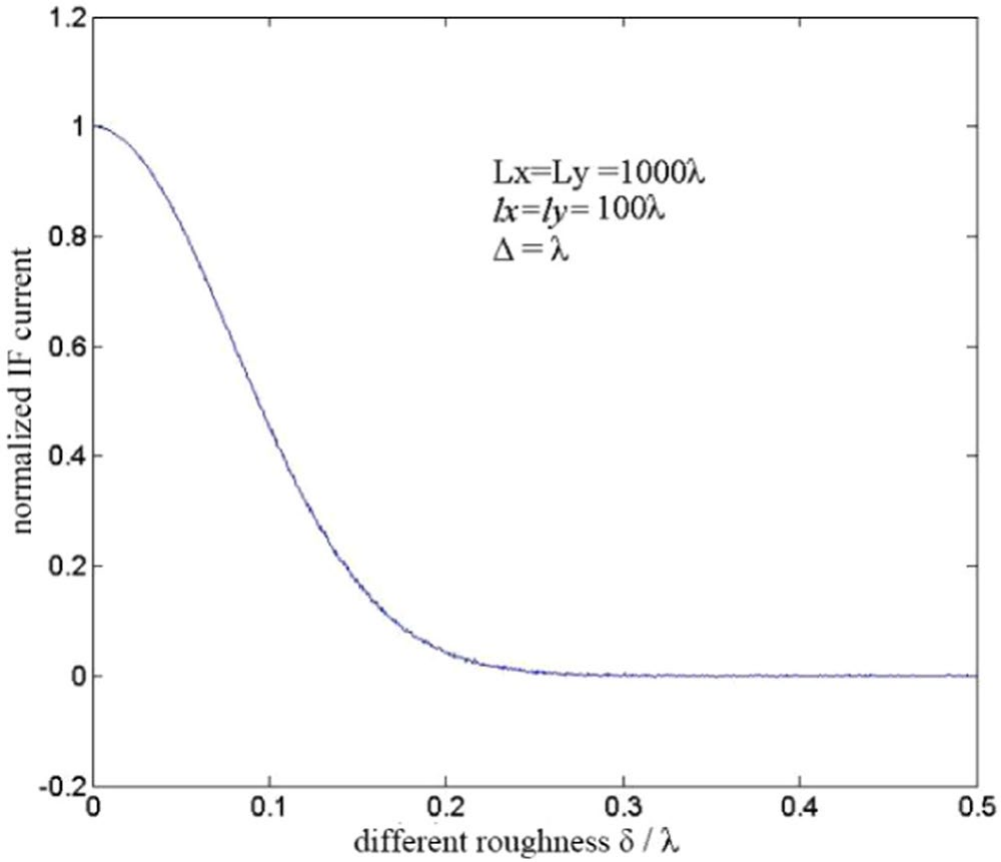
Normalised IF current variation with the root mean square of roughness height.

The simulation wavelength was set to 532 nm in this study. The relevant length was constant (*l*_*x*_ = *l*_*y*_ = 100 *λ*), sampling length was 1000 *λ* (*L*_*x*_ = *L*_*y*_ = 1000 *λ*), and number of discrete points per rough surface was 1000 × 1000. The root mean square was *δ* ∈ (0,2 *λ*), and the step length was 0.01 *λ*; we counted 1000 times the IF currents for each *δ* current, and then took the average, normalised IF current. The normalised IF current, with the height fluctuation of the root mean square variation of the relationship, is shown in Fig. 4. The equation used for generating y-axis of Fig. 4 is:

15 $${i}_{IF}=\frac{{i}_{IFO}}{N}\mathop{\sum }\limits_{i=1}^{N}\cos \left({\omega }_{IF}t+\frac{4\pi }{\lambda }{h}_{i}\right),$$

where i_IFO_ indicates the total intermediate frequency current received by the detector when target surface is smooth, N indicates the photosensitive surface of detector is divided into N uniform small surface elements, h_i_ is the height fluctuation of random rough surface.

When 0 < *δ* < 0.2*λ*, the normalised IF current decreases as x increases. When *δ* > 0.2*λ*, there is no evident law, and the IF frequency is near zero. In addition, when the target surface is sufficiently rough, the heterodyne signal is weak.

To compensate for this performance degradation, we developed an efficient signal processing method using the following process.

For convenience, we redefine the in-phase IF signal as shown in (15):

16 $${i}_{I}(t)=\frac{1}{A}{\iint }_{A}\cos [\Omega t+{\varphi }_{S}(r)]ds,$$

where *i*_*i*_*(t)* denotes the IF current produced per unit area of the photodetector. The orthogonal IF component can be obtained through a 90° phase shift, as shown in Eq. (16).

17 $${i}_{Q}(t)=-\frac{1}{A}{\iint }_{A}\sin [\Omega t+{\varphi }_{S}(r)]ds.$$

By using an integral operation, we can introduce two signals, *B*_*X*_ and *B*_*Y*_, which are defined as shown in Eq. (17) and (18).

18 $${B}_{X}=\frac{1}{T}{\int }_{0}^{T}{i}_{I}(t)\cos \,\Omega tdt=\frac{1}{2}X,$$

19 $${B}_{Y}=\frac{1}{T}{\int }_{0}^{T}{i}_{Q}(t)\sin \,\Omega tdt=\frac{1}{2}Y.$$

where T = 2π/Ω and

20 $$X=\frac{1}{A}{\iint }_{A}\cos {\varphi }_{S}(r)ds,$$

21 $$Y=\frac{1}{A}{\iint }_{A}\sin {\varphi }_{S}(r)ds.$$

The modified IF current is represented by *I*_*IF*_(*t*), which is defined as

22 $$\begin{array}{rcl}{I}_{IF}(t) & = & {B}_{X}{i}_{I}(t)-{B}_{Y}{i}_{Q}(t)\\  & = & \frac{1}{2}U\,\cos \,\Omega t\end{array}$$

where *U* = *X*^2^ + *Y*^2^. The amplitude U is the sum of two independent squared normal random variables. Therefore, U has an exponential distribution characterised by

23 $$P(U)=\frac{1}{\langle U\rangle }\exp \left(-\frac{U}{\langle U\rangle }\right)$$

where 〈*U*〉  = 2 *σ*². Thus, the expectation of *I*_*IF*_(*t*) is

24 $$\langle {I}_{IF}(t)\rangle ={\sigma }^{2}\,\cos \,\Omega t.$$

Equation (24) indicates that the modified IF current contains a non-zero IF component, indicating that the decoherence effect has been compensated. Equivalently, the phase fluctuation, *φ*_*s*_ (*r*), in the integrand in Eq. (4) is effectively removed, because Eq. (21) is not dependent on *φ*_*s*_ (*r*). The compensation method only acts on a random phase. If *φ*_*s*_ (*r*) = *φ*_0_ is a constant, we have X = cos *φ*_0_ and Y = sin *φ*_0_. From Eq. (22), the following expression can be obtained:

25 $${I}_{IF}(t)=\frac{1}{2}\,\cos \,\Omega t.$$

This result only contains the IF component, implying that the compensation algorithm does not act on an IF signal without a random phase distribution.

**Figure 5:**
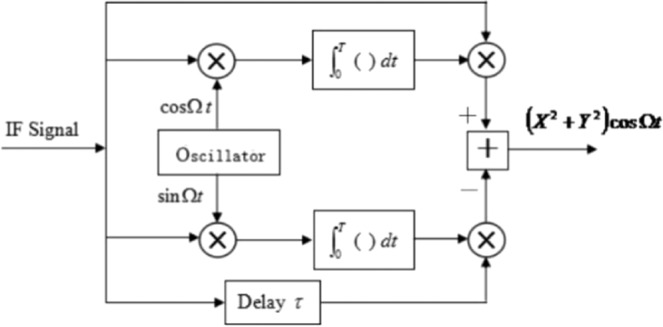
Block diagram of the IF signal processing system for compensating the decoherence effect; the upper limit of the integrals is T = 2π/Ω.

According to the compensation principle presented above, a block diagram of the proposed compensation algorithm can be given as shown in Fig. 5.

The IF signal, i_IF_(*t*), from a traditional heterodyne receiver (single branch detection or balanced detection) is divided into four channels. Channel 1 generates a signal *i*_*I*_(*t*), and Channel 4 generates a signal *i*_*Q*_(*t*) with a 90° phase shift. Channels 2 and 3 are mixed using cosine and sine signals, respectively. The two mixed signals generate *B*_*x*_ and *B*_*y*_ signals, using two integrators, respectively. Next, *B*_*x*_ is mixed with *i*_*I*_(*t*), *B*_*y*_ is mixed with *i*_*Q*_(*t*), and the two mixed signals are given as inputs for the adder, which outputs the modified IF signal. If a light signal is divided into four channels, the signal power for each channel will reduce to ¼ of the original signal power, which can degrade the sensitivity. Normally, a CMOS device is a voltage-dependent device with extremely high input impedance. Therefore, the division of an electric signal into four channels cannot result in attenuation.

According to the principle of the proposed compensation method, the compensating effect depends on the stability of the oscillator frequency, which should track the intermediate frequency exactly.

Phase variations in the output signal of the oscillator in Fig. 5 can lead to an unstable IF signal, *I*_IF_(*t*). Phase stability of the oscillator can be obtained by using a phase-locked loop, and a reference signal can be extracted directly from the input IF signal.

## Experiments

### Compensating for decoherence effect in heterodyne detection caused by rough targets

**Figure 6:**
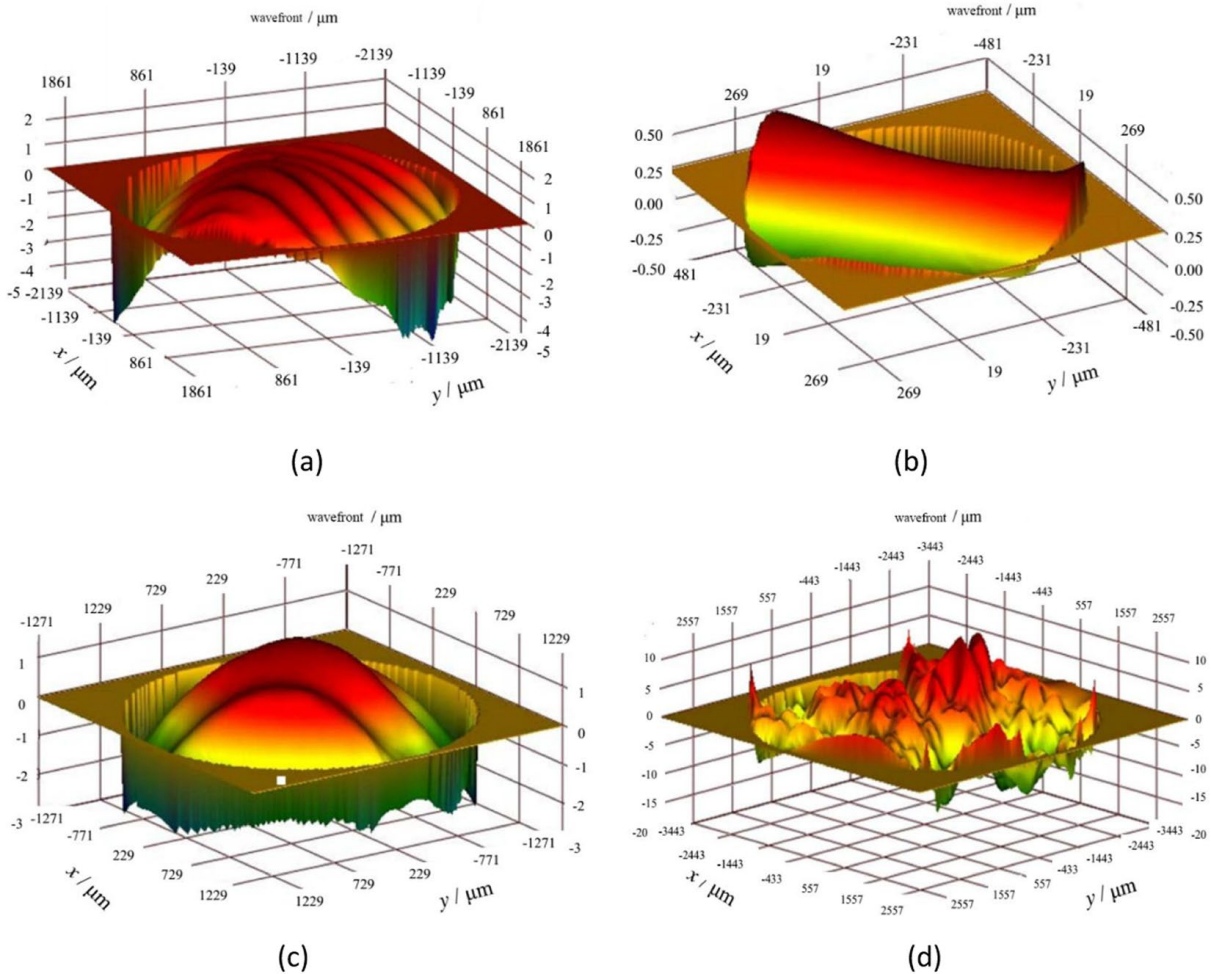
Wavefront at different locations: (**a**) light wavefront; (**b**) LO light wavefront; (**c**) reflector echo wavefront; (**d**) wavefront for printed paper.

We first measured the LO light and signal light wavefronts as indicated in Fig. 1, and then used a wavefront analyser to measure the wavefront of the laser source, as shown in Fig. 6(a). The wavefront analyser was then placed on the photo-sensor surface of the photodetector to measure the front of the LO light wave, as shown in Fig. 6(b).

When measuring the signal light wavefront, the wavefront analyser was placed on the photo-sensing surface of the photodetector and LO, blocking the local oscillation light was blocked during the measurement process. The detection target was a plane mirror, indicating that the target was smooth, and the signal light wavefront was measured, as shown in Fig. 6(c). Then, with the target and the measurement position unchanged, the target was changed to a piece of printer paper representing a rough surface. The resultant signal light wavefront measurement is shown in Fig. 6(d).

The wavefronts for the LO light wave and mirror echo signal are slightly undulating and relatively smooth. However, there is no regularity in the surface undulations of the printer paper echo signal wavefront.

Wavefront matching is very difficult and it significantly affects the heterodyne signals.

**Figure 7:**
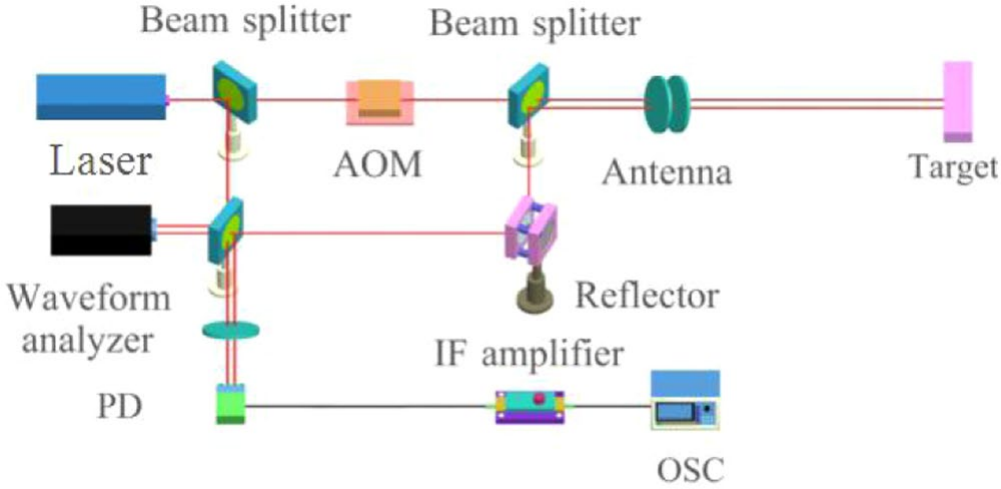
Schematic of the heterodyne detection system.

The proposed heterodyne detection system is illustrated in Fig. 7. The main feature of our system is that a digital wavefront analyser—that can obtain the actual phase wavefront and the heterodyne signal simultaneously is added to a traditional heterodyne system.

**Figure 8:**
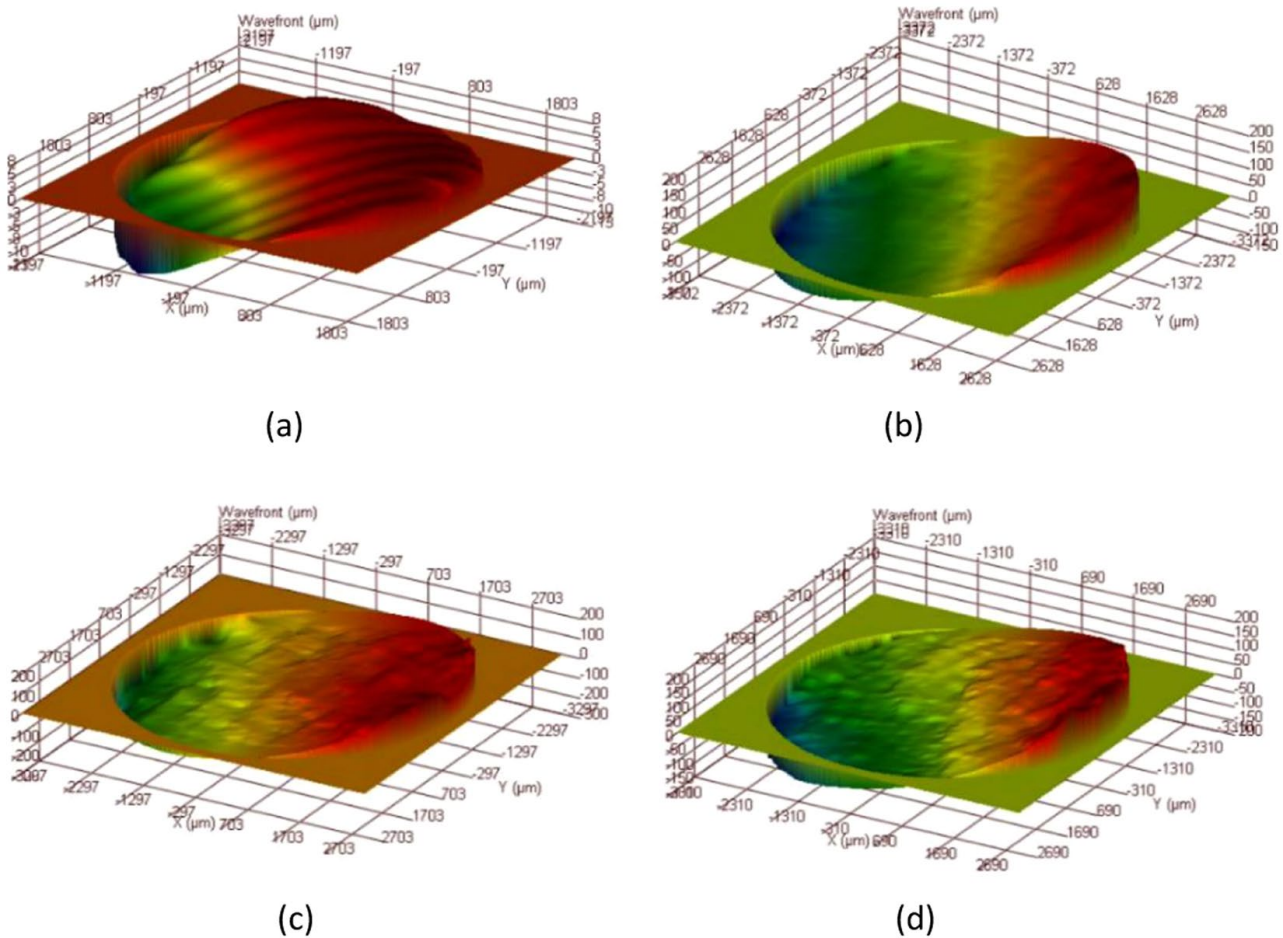
Actual beam echo wavefronts for surfaces with different degrees of roughness. (**a**) *δ* = 0 *λ*, (**b**) *δ* = 0.1 *λ*, (**c**) *δ* = 0.2 *λ*, and (**d**) *δ* = 0.4 *λ*.

We used a Verdi-II laser with a wavelength of 532 nm and linewidth of 5 MHz as a light source. Using a digital waveform analyser, the actual wavefront beams of the rough surface target were tested. The results are shown in Fig. 8.

By comparing Figs. 3 and 8, one can see that the simulation results are very consistent with the experimental results. Additionally, the phase of the laser beam is deeply modulated by the rough target surface.

**Figure 9:**
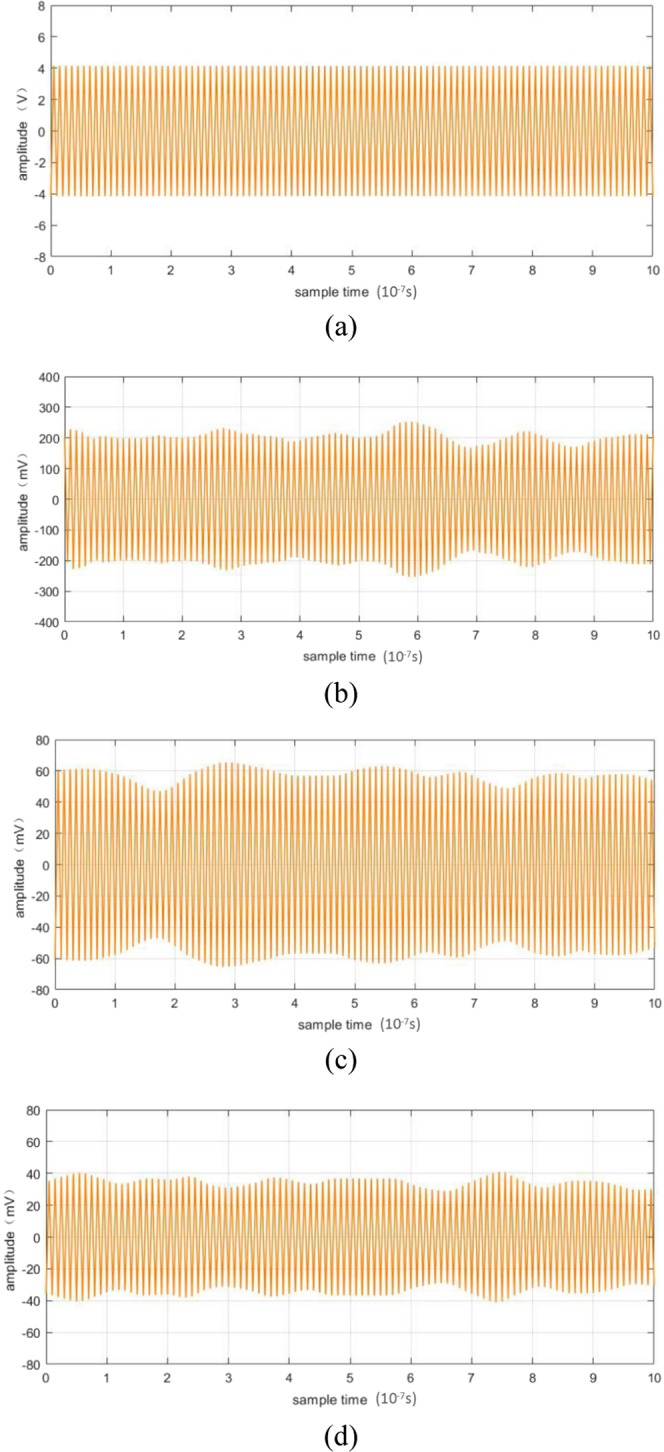
Heterodyne signals obtained for surfaces with different degrees of roughness, under the same LO and signal optical power conditions. (**a**) *δ* = 0 *λ*, (**b**) *δ* = 0.1 *λ*, (**c**) *δ* = 0.2 *λ*, and (**d**) *δ* = 0.4 *λ*.

Heterodyne signals for surfaces with different degrees of roughness were also measured. The results depicted in Fig. 9. Figure 9(a–d) present heterodyne signals obtained for surfaces with different degrees of roughness under the same LO and continuous source conditions.

According to Eqs. (5), (6), and (7), Fig. 9 presents the variations in heterodyne efficiency 〈i_*IF*_(*t*)〉 with the root mean square *δ*. In comparison to Fig. 9(a,d), the heterodyne signal attenuated by 20 dB, owing to the roughness of the target surface. For this single-detector system, intensity decreases significantly with an increasing value of the root mean square *δ*.

**Figure 10:**
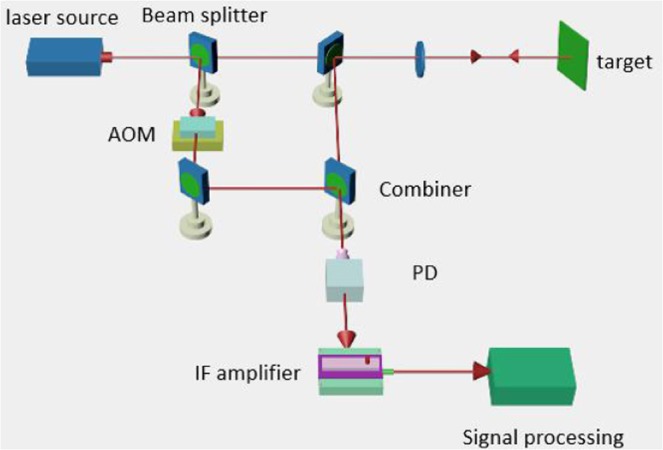
Schematic of the experimental setup.

To test the performance of the proposed compensation technique, we constructed an optical heterodyne detection system for a rough target, using the experimental setup shown in Fig. 10. The signal processing unit includes the compensation system shown in Fig. 5.

In our experiments, a Coherent Inc. Verdi-II laser with a 532 nm wavelength and 5 MHz linewidth was used as a light source. We also used a LeCroy WaveSurfer 62Xs oscilloscope with a sample rate of 2.5 GS/s. The laser output was divided into two parts by a beam splitter. One part was sent to the transmission optics to illuminate the target, and the other was sent into an acousto-optic (AO) modulator, which was used to produce a 100 MHz frequency shift in the laser. The output of the AO modulator, which acts as the LO for coherent heterodyne detection, was then sent directly to the PD. The target was a piece of printer paper that was sufficiently rough in comparison to the wavelength.

A variable-gain IF amplifier with a 20 MHz/3 dB bandwidth was used. IF signal was sampled using an analogue-to-digital converter and stored. Digital signal processing including multiplication, addition, and integration was performed using a desktop computer. The necessary 90° phase shift in the IF signal was obtained by applying a shift operation to the IF data in memory.

**Figure 11:**
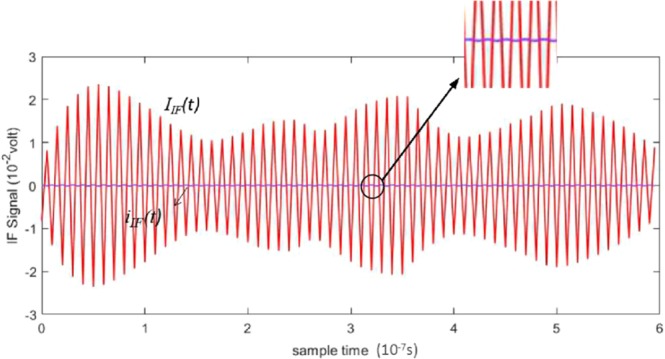
Comparison between IF signals received with and without application of the decoherence effect compensation technique.

Figure 11 shows a comparison between the IF signals of heterodyne receiver with and without the proposed compensation technique. The gain of the IF amplifier and the experimental setup are identical in both cases. The IF signal i_*IF*_(*t*) is difficult to observe because its amplitude is only approximately 10^−4^ V, while the amplitude of *I*_*IF*_(*t*) is as high as 2.4 × 10^−2^ V. This demonstrates that the sensitivity of the detection system is significantly improved by the proposed compensation technique.The signal-to-noise ratio of the system is improved by 47.6 dB.

This experiment demonstrated that the proposed compensation algorithm can reduce the difficulty of optical experiments and can easily be implemented in the electrical field. The proposed algorithm ensures that the modified IF current contains non-zero IF components. As shown in Fig. 11, the amplitude of the compensated signal is approximately 200 times the amplitude of the signal without compensation, indicating that the decoherence effect is effectively mitigated. However, due to long distance, there are many factors that affect experimental results. Therefore, our method is not very effective in detecting large target at long distance.

#### Target vibration characteristic measurement

**Figure 12:**
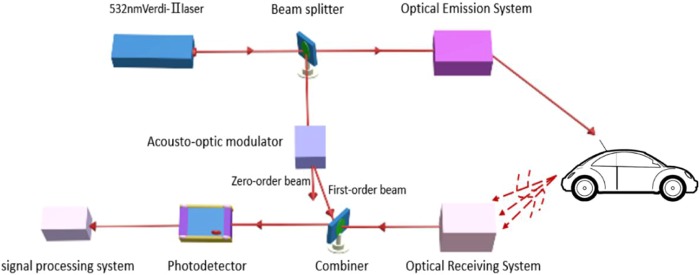
Schematic of the automobile vibration measurement system.

According to various studies, targets powered by engines, such as airplanes, automobiles, and ships, vibrate at a certain frequency. When detecting a target, vibration information is captured by optical signals, meaning a target can be identified according to the vibration spectra in echo signals. Based on reported experimental results, it can be concluded that each type of target has a specific vibration spectrum related to the nature of the corresponding engine (e.g., shape, power, and mass), providing unique identification characteristics similar to human fingerprints. Therefore, the vibration characteristics of a target can be used as features for target recognition. A schematic of a vehicle vibration measurement system is presented in Fig. 12.

This system contains five components: a light source, an optical transmitting system, an optical receiving system, a signal processing system, and a target. A Verdi-II laser with a wavelength of 532 nm, a linewidth of 5 MHz, and an output power of 1 W was used as a light source. The optical emission system includes a beam expander and reflection mirrors. The beam divergence angle is less than 0.5 mrad, giving a transmission strength greater than 96%. A Thorlabs Co. AC508-101-A mirror with a receiver aperture of 2 mm is used as an optical receiving system and a GT106 PIN device that can receive power on the order of microwatts is used as a photodetector. The signal processing system includes the compensation system presented in Fig. 5.

We pasted A4 printer paper onto the vehicle’s air intake grille to create a diffuse surface. The target was a vehicle manufactured by Citroen. During measurement, the vehicle engine was in an idle state at approximately 800 rpm and the front of the vehicle was located 20 m from the optical measurement system. The laser transmitting system, receiving system, AO modulator, amplifier, filter, and detector were all fixed on the test platform.

**Figure 13:**
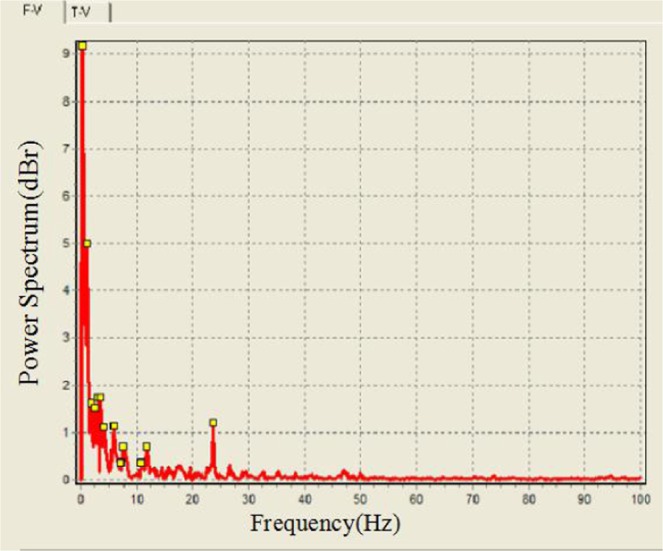
Vehicle vibration spectrum peak point analysis.

The automotive vibration power spectrum harmonics and peak points captured during this experiment are presented in Fig. 13.

The peak data in Fig. 13 are listed in Table 1.

**Table 1:** Vehicle vibration spectrum peak points.

Peak point	1	2	3	4	5	6	7
Frequency (Hz)	0.25	1	1.75	2.5	3	3.5	4
Peak point	8	9	10	11	12	13	
Frequency (Hz)	5.75	7	7.5	10.75	11.75	23.5	

The experiments described above revealed successful detection of the spectrum of vehicle vibration. This is considered to be an important result in terms of laser Doppler target recognition based on heterodyne technology. Additionally, these results have established a foundation for future research.

## Conclusion

In most cases, both the phase and amplitude of a laser beam are modulated by rough target surfaces during heterodyne detection. A heterodyne signal can be considered as a cosine signal. However, the addition of cosine functions with different phases decreases its amplitude. Furthermore, the decoherence effect decreases the sensitivity of heterodyne detection significantly, even when 〈i_*IF*_(*t*)〉 = 0. Compared with a smooth surface, a rough surface with a root mean square value of 0.4 µm causes heterodyne signal attenuation of 20 dB.

In this study, an effective method for compensating for the decoherence effect using mature signal processing techniques in the electric domain. The proposed scheme can be easily implemented using modern digital signal processors. Additionally, the proposed method can provide new technical insights into mitigating the decoherence effect in active heterodyne measurement systems. This technique can also compensate for any decoherence effects caused by atmospheric turbulence, provided that the variations in turbulence over time are not severe compared with the intermediate frequency.

The proposed vehicle vibration measurement system is also suitable for measuring the vibration spectra of various target types, such as other vehicle types, aircraft, and ships. The proposed system establishes a foundation for the application and popularisation of laser Doppler detection and has expanded our understanding of the technique.

## Supplementary information

Supplementary information.
