# Supplementary material for: Compensation for the Decoherence Effect in Heterodyne Detection of Rough Targets and a Target Vibration Characteristic Measurement System
Source: Sci Rep. 2020 Apr 8;10:6077. doi: 10.1038/s41598-020-62966-0 (PMC7142099; doi:10.1038/s41598-020-62966-0)
Supplement: Supplementary file 1 — Supplementary information. [file 41598_2020_62966_MOESM1_ESM.pdf]

Compensation for the Decoherence Effect in Heterodyne Detection of Rough Targets and a Target Vibration Characteristic Measurement System

Changqing Cao[1], Xiyuan Su*[1], Yutao Liu[1], Xiaodong Zeng[1], Zhejun Feng[1], Jingshi Shen[2], Ting Wang[1] and Xu Yan[1]

[1] School of Physics and Optoelectronic Engineering, , 2 South Taibai Road, Xian, 710071, China.
[2] Shandong Institute of Space Electronic Technology, Yantai, 264670, China
*erinsxy@163.com

We define the in-phase IF signal as follow：

$$i_I(t) = \frac{1}{A}\iint_A \cos[\Omega t + \varphi_s(r)]ds\,, \tag{1}$$

The orthogonal IF component can be obtained through a 90° phase shift, as shown in Eq (2).

$$i_Q(t) = -\frac{1}{A}\iint_A \sin[\Omega t + \varphi_s(r)]ds\,. \tag{2}$$

we introduce two signals B_X and B_Y , defined as：

$$B_X = \frac{1}{T}\int_0^T i_I(t)\cos\Omega t dt\,, \tag{3}$$

$$B_Y = \frac{1}{T}\int_0^T i_Q(t)\sin\Omega t dt\,. \tag{4}$$

Input $i_I(t)$ into B_X, we can get:

$$B_X = \frac{1}{T}\int_0^T \frac{1}{A}\iint_A \cos[\Omega t + \varphi_s(r)]\cos\Omega t ds dt\,. \tag{5}$$

$$B_X = \frac{1}{AT}\int_0^T \frac{1}{A}\iint_A \frac{1}{2}\{\cos[\Omega t + \varphi_s(r)] + \cos\varphi_s(r)\}ds dt\,. \tag{6}$$

$$B_X = \frac{1}{2AT}\int_0^T \left[\iint_A \cos[2\Omega t + \varphi_s(r)]ds + \iint_A \cos\varphi_s(r)ds\right]dt\,. \tag{7}$$

$$B_X = \frac{1}{2A}\iint_A \cos\varphi_s(r)ds = \frac{1}{2}X \tag{8}$$

$$X = \frac{1}{A}\iint_A \cos\varphi_s(r)ds \tag{9}$$

the same reason,

$$B_Y = -\frac{1}{2A}\iint_A \cos\varphi_s(r)ds = \frac{1}{2A}\iint_A \sin\varphi_s(r)ds = \frac{1}{2}Y \tag{10}$$

$$Y = \frac{1}{A}\iint_A \sin\varphi_s(r)ds \tag{11}$$

The modified IF current is represented by $I_{IF}(t)$, defined as:

$$I_{IF}(t) = B_X i_I(t) - B_Y i_Q(t) \tag{12}$$

At first we can get:

$$B_X i_I(t) = \frac{1}{A}\cdot\iint_A \cos[\Omega t + \varphi_s(r)]ds \cdot \left[\frac{1}{2A}\cos\varphi_s(r)ds\right] \tag{13}$$

$$B_X i_I(t) = \frac{1}{2A^2}\left\{\left[\iint_A \cos\Omega t\cos\varphi_s(r) - \sin\Omega t\sin\varphi_s(r)ds\right]\cdot\iint_A \cos\varphi_s(r)ds\right\} \tag{14}$$

$$B_X i_I(t) = \frac{1}{2A^2}\left\{\left[\cos\Omega t\iint_A \cos\varphi_s(r)ds - \sin\Omega t\iint_A \sin\varphi_s(r)ds\right]\cdot\iint_A \cos\varphi_s(r)ds\right\} \tag{15}$$

$$B_X i_I(t) = \frac{1}{2A^2}\left\{\cos\Omega t\left(\iint_A \cos\varphi_s(r)ds\right)^2 - \sin\Omega t\iint_A \sin\varphi_s(r)ds\cdot\iint_A \cos\varphi_s(r)ds\right\} \tag{16}$$

the same reason,

$$B_Y i_Q(t) = -\frac{1}{2A^2}\left\{\cos\Omega t\left(\iint_A \sin\varphi_s(r)ds\right)^2 + \sin\Omega t\iint_A \sin\varphi_s(r)ds\cdot\iint_A \cos\varphi_s(r)ds\right\} \tag{17}$$

therefore

$$I_{IF}(t)=\frac{1}{2A^2}\cos\Omega t\left[\left(\iint_A \cos\varphi_s(r)ds\right)^2+\left(\iint_A \sin\varphi_s(r)ds\right)^2\right] \quad (18)$$

$$I_{IF}(t)=\frac{1}{2}U\cos\Omega t \quad (19)$$

where $U=X^2+Y^2$, X and Y follow uniform distribution as follows:

$$f_X(x)=\frac{1}{\sqrt{2\pi\sigma^2}}e^{-\frac{x^2}{2\sigma^2}} \quad (20)$$

$$f_Y(y)=\frac{1}{\sqrt{2\pi\sigma^2}}e^{-\frac{y^2}{2\sigma^2}} \quad (21)$$

$$f_{XY}(xy)=\frac{1}{2\pi\sigma^2}e^{-\frac{x^2+y^2}{2\sigma^2}} \quad (22)$$

The distribution function of U is shown in Eq. (23):

$$F_U(u)=\iint_{x^2+y^2\le u}\frac{1}{2\pi\sigma^2}e^{-\frac{x^2+y^2}{2\sigma^2}}dxdy \quad (23)$$

$$F_U(u)=\int_0^{2\pi}\int_0^{\sqrt{u}}\frac{1}{2\pi\sigma^2}e^{-\frac{r^2}{2\sigma^2}}rd\theta dr=1-e^{-\frac{u}{2\sigma^2}} \quad (24)$$

$$f_U(u)=F_U'(u)=\frac{1}{2\sigma^2}e^{-\frac{u}{2\sigma^2}} \quad (25)$$

$\langle U\rangle=2\sigma^2$, $\langle\cdot\rangle$ denotes the average over the ensemble of realizations. Thus U has an exponential distribution and is characterized by:

$$P(U)=\frac{1}{\langle U\rangle}\exp\left(-\frac{U}{\langle U\rangle}\right) \quad (26)$$

Finally we can get that

$$I_{IF}(t)=\frac{1}{2}\cos\Omega t \quad (27)$$
